# A Review on Chitosan and Cellulose Hydrogels for Wound Dressings

**DOI:** 10.3390/polym14235163

**Published:** 2022-11-27

**Authors:** Collins N. Elangwe, Svetlana N. Morozkina, Roman O. Olekhnovich, Alexander Krasichkov, Victoriya O. Polyakova, Mayya V. Uspenskaya

**Affiliations:** 1 Chemical Engineering Center, ITMO University, Kronverkskiy Prospect, 49A, Saint Petersburg 197101, Russia; 2 Departments of Radio Engineering Systems, Electrotechnical University “LETI”, Prof. Popova Street 5F, Saint Petersburg 197022, Russia; 3 St. Petersburg Research Institute of Phthisiopulmonology, Ligovsky 2-4, Saint Petersburg 191036, Russia

**Keywords:** chitosan, cellulose nanocrystals, hydrogels, wound dressing, chronic wounds, market products

## Abstract

Wound management remains a challenging issue around the world, although a lot of wound dressing materials have been produced for the treatment of chronic and acute wounds. Wound healing is a highly dynamic and complex regulatory process that involves four principal integrated phases, including hemostasis, inflammation, proliferation, and remodeling. Chronic non-healing wounds are wounds that heal significantly more slowly, fail to progress to all the phases of the normal wound healing process, and are usually stalled at the inflammatory phase. These wounds cause a lot of challenges to patients, such as severe emotional and physical stress and generate a considerable financial burden on patients and the general public healthcare system. It has been reported that about 1–2% of the global population suffers from chronic non-healing wounds during their lifetime in developed nations. Traditional wound dressings are dry, and therefore cannot provide moist environment for wound healing and do not possess antibacterial properties. Wound dressings that are currently used consist of bandages, films, foams, patches and hydrogels. Currently, hydrogels are gaining much attention as a result of their water-holding capacity, providing a moist wound-healing milieu. Chitosan is a biopolymer that has gained a lot of attention recently in the pharmaceutical industry due to its unique chemical and antibacterial nature. However, with its poor mechanical properties, chitosan is incorporated with other biopolymers, such as the cellulose of desirable biocompatibility, at the same time having the improved mechanical and physical properties of the hydrogels. This review focuses on the study of biopolymers, such as cellulose and chitosan hydrogels, for wound treatment.

## 1. Introduction

Wound can be defined as a damage of living skin or tissue [1]. According to various injury factors, wounds are known as: bruises, incisions, injuries, and cuts. It is mostly caused by external injury factors, namely surgery, external force, heat, current, chemicals, low temperature, and by internal factors such as local blood supply disorders. Wounds are classified as chronic and acute. Acute wounds can heal within 60–90 days depending on the nature (depth and size) of the wound [2]. Chronic wounds are wounds that heal significantly slower and fail to progress to all the phases of the normal wound heal process and are usually stalled at the inflammatory phase [3]. These wounds cause a lot of challenges to patients such as severe emotional and physical stress and generate a considerable financial burden on patients and the general public healthcare system. It has been documented that about 1–2% of the global population suffers from chronic non-healing wounds during their lifetime in developed nations. Traditional wound dressings are dry, and therefore cannot provide moist environment for wound healing and do not possess antibacterial properties [4]. Wound dressings that are presently used include films, foams, bandages, patches, and hydrogels. Nowadays, hydrogels are gaining a lot of attention as a result of their water-holding capacity, providing a moist wound milieu. Chitosan is a biopolymer that has received great attention recently in pharmaceutical industries because of its unique chemical and antibacterial nature. However, with its poor mechanical properties, chitosan is incorporated with other biopolymers to take advantage of desirable biocompatibility of chitosan at the same time having the improved mechanical and physical properties of the hydrogels. Naturally derived biomaterials such as carbohydrates have been employed to improve the mechanical properties of hydrogels. Cellulose is a highly abundant natural polymer which continues to attract a lot of attention until now because it is easily available, biodegradable and non-toxic [5,6]. Cellulose is usually incorporated with other polymers because it has a large surface area, non-toxic, excellent mechanical properties, biodegradable, and low density [7]. Although there are huge number of investigations based on the development of hydrogels from cellulose in various applications, the reinforcement of chitosan with cellulose materials in wound dressing continues to be of great interest. Furthermore, the encapsulation of therapeutic agents such as antibiotics, antioxidants, and growth factors and cells in hydrogels will enhance wound healing.

Hydrogels are three-dimensional network of cross-linked hydrophilic polymers which have the ability to absorb large volumes of water (water content can be up to 99%) [6,8,9]. The swelling ability of hydrogels is due to hydrophilic groups (-OH, -CONH-, -CONH_2_, and -SO_3_H) present in the polymeric components of the gels [10]. Hydrogels are derived from natural and synthetic polymers via physical or chemical crosslinking. The high-water content of hydrogels makes them compatible with most living tissues and thus facilitates widespread application in biomedical and pharmaceutical fields. For the past few years, investigators have focused their attention on the search for non-toxic and biocompatible materials for living organisms [11]. Over the past years, hydrogels have been used as drug delivery systems [12], wound dressings [9,13] gene transfection [14,15], tissue engineering scaffolds [16,17], and biosensors [18].

## 2. Wound Healing Phases

**Figure 1:**
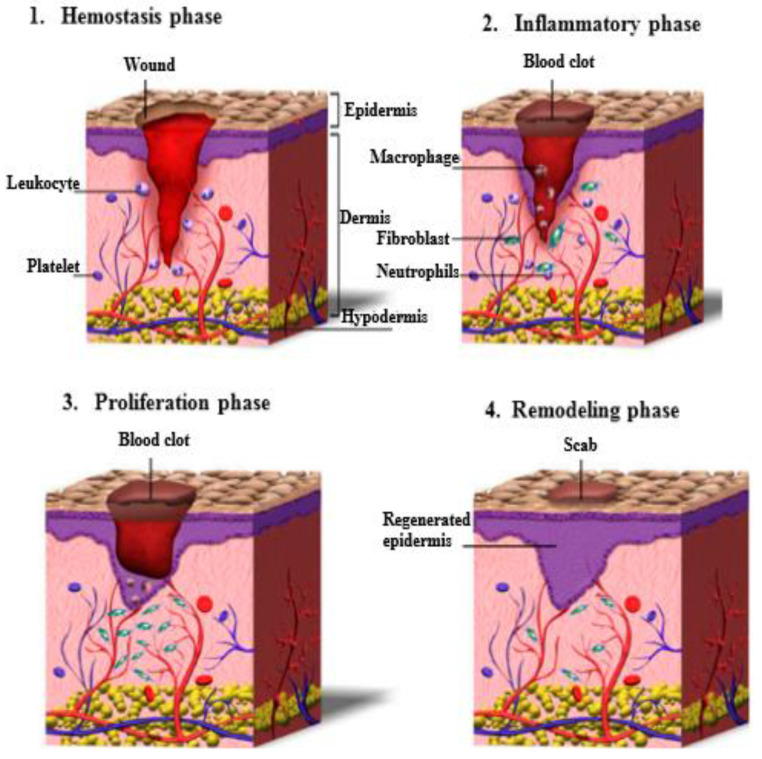
Schematic presentation of the wound healing process. Reproduced from [20], with permission from MDPI, 2022.

Wound healing is a highly dynamic and complex regulatory process that involves four principal integrated phases, including hemostasis, inflammation, proliferation and remodeling [19,20,21,22] as illustrated in Figure 1. These four phases have to begin in a well-defined sequence and should last for a certain period, and there can be a partially comprehensive overlap between the phases.

Hemostasis: the objective of the hemostasis phase is to stop bleeding. In this phase the body activates its blood clotting systems. Hemostasis comprises of vascular constriction and platelet activation, following their interaction with the extracellular matrix and damaged collagen fibers. The formation of fibrin network produces a clot, which is a temporary matrix that provides strength to the injured tissues and supports cell migration [22,23,24]. When the blood clots at the opening of a wound, it prevents the body from losing too much blood and it is the first step of wound closure [25]. This stage can last up to two days depending on how deep the wound is.

Inflammation: once phase one is completed and the body has stopped bleeding, the body activates its key defense mechanism—inflammation. This stage works to kill bacteria and remove debris with white and other blood cells. In the inflammatory phase, immune cells (particularly neutrophils and macrophages) infiltrate into the wound where they phagocyte damaged and dead cells, bacteria, and other pathogens or debris [20,22,26]. In addition, inflammatory cells and platelets release several peptide growth factors, promoting the migration of fibroblasts into the injury site and activating angiogenesis [20]. Inflammation ensures that the wound is clean and ready for new tissue to start growing.

Proliferation: the cell proliferation phase involves re-epithelialization, angiogenesis, and granulation tissue formation, which is the second temporary matrix containing fibroblasts and macrophages [20,26]. During this stage, the fibroblasts produce collagen and the myofibroblasts will promote the process of wound edges contraction [23]. This phase can be divided into three sub-phases, including: (1) filling the wound with new connective tissues and blood vessels, (2) contracting the edges of the wound: tightening the wound, (3) covering the wound: epithelial cells that form a protective barrier between the inside and outside of the body migrate into the wound to close the wound completely.

Remodeling: during wound remodeling (also called maturation phase), the excess collagen fibers in the wound are broken down in the dermis, and contraction of wound starts to reach its maximum. Fibroblasts control the degradation of the wound matrix via the formation of matrix metalloproteinases (MMPs) and new cellular connective tissues [27]. At this point, the repaired wound attains its maximum mechanical strength. The final scar will have 80% of the original strength of the wound [20,28].

## 3. Types of Wounds

Wounds are of different types, which are caused as a result of physical, chemical, and thermal damages. Depending on the nature of the healing process, wounds can be divided into two main types, namely acute and chronic wounds [6,20].

Acute wound: an acute wound is an injury to the skin that takes place immediately rather than over time. Acute wounds in a normal healthy person will heal fast at the rate of the normal wound healing process because of a balance of growth factors, cytokines, and matrix metalloproteinase (MMPs) [29]. Basically, acute wounds can occur on any part of the body, which can range from superficial bruises to deep wounds causing damage to blood vessels, nerves, and muscles. Acute wounds may last up to 2 to 3 months followed by infection, pain, and necrosis [30]. Some examples of acute wound include (i) surgical wounds: Surgical wounds are incisions made intentionally by a medical professional and are cut precisely, creating clean edges around the wound. Surgical wounds may be closed (with stitches, staples or adhesive) or left open to heal by primary intention, (ii) traumatic wounds: These are unplanned injuries that can range from minor injuries such as a skinned knee, to severe injuries such as a gunshot wound. Examples of traumatic wounds consist of abrasions, skin tears, bites, and penetrating trauma wounds, (iii) burns: A burn is a type of injury to skin or other tissues caused by heat, cold, electricity, chemical, friction or radiation.

Chronic wound: it is a wound that fails to heal in a well-ordered set of stages and in an expected period of time of normal wound healing process. Wounds that take a long time (that is more than 90 days) to heal are generally considered chronic. Chronic wounds sometimes do not proceed to one or more of the wound healing phases. For example, chronic wounds are often stalled at the inflammatory phase for too long a period of time. Some of the common types of chronic wounds are diabetic foot ulcers, venous and arterial ulcers, and pressure ulcers [6,31]. Chronic wounds may take a very long period to heal or may never heal. These wounds cause severe emotional and physical stress, and pain in patients. Many factors are usually responsible for wound impairment. This is as a result of overlapping mechanisms in normal wound healing process that tends to prevent one factor from disrupting the process. However, when the healing process is disrupted and wound healing is impaired, this will lead to the development of chronic wounds. Generally, the main factors affecting chronic non-healing wounds include infection, imbalance in matrix metalloproteinases and matrix metalloproteinases inhibitors, oxidative stress, metabolic conditions, immunosuppression, and radiation.

Bacterial infection in wounds is the most often reason of the wound healing process interruption. Bacteria generate inflammatory markers that prevent the inflammatory phase as well as epithelialization phase of wound healing. The presence of bacteria in an infected wound leads to cell death, which causes an increase in inflammation response and persistent inflammatory phase. Necrotic tissues present in wounds disrupts the ingrowth of new tissues. In addition, necrotic tissue also serves as a ground for bacterial growth, leading to a pathologic cycle. When the bacterial burden of a chronic wound is more than 1 × 10^6^ colony forming units per gram of tissue, it is considered as being clinically infected [32]. Commonly encountered, chronic wound bacteria include *Staphylococcus aureus*, *Pseudomonas aeruginosa*, *Enterococcus faecalis*, *Proteus* spp., *Streptococcus* spp., *Escherichia coli*, *Citrobacter* spp., *Morganella* spp. and *Corynebacterium* spp. Bacteria form protective biofilms that are not recognized by the host cells. Biofilms severely affect the wound healing process because they disrupt the immune response, prolong epithelialization, and decrease the growth of granulation tissues.

Persistent oxidative stress in chronic wounds disrupt inflammatory responses resulting in poor angiogenesis and re-epithelialization is impaired [33]. Oxidative stress is as a result of excess reactive oxygen species (ROS) production in the wound. ROS consist of hydrogen peroxide H_2_O_2_, superoxide anion O_2_^−^ or peroxide O_2_^2−^. They are powerful oxidants and contribute enormously to cell damage, but they also play a vital role in the preparation of the normal healing process. Therefore, a balance between low and high level of ROS is very important. Low levels of reactive oxygen species are essential in the protection of tissues against bacterial infection and promoting wound healing by the production of cell surviving signaling [34]. There is no clear cut-off point for reactive oxygen species level in tissues but for normal wounds, the level of hydrogen peroxide (which is the most common oxidant) is in the range 100–250 µM [34].

## 4. Hydrogels as Biomaterials

Biomaterial is any material (synthetic or natural) used as a complete or as part of a biological system which has been impaired or to interact with living systems for medical purposes [35,36]. Biomaterial should be compatible and biodegradable. Biomaterials should not possess any kind of unfavorable or side reaction from the living tissue and vice versa. The biomedical applications of biomaterials include hip joints, drug carrier devices, bone plates, contact lenses, wound dressings [35]. Biomaterials such as gelatin, alginate, hyaluronic acid, dextran, elastin, collagen, cellulose nanocrystals, chitosan have gained great interest and are widely used for wound dressings and as drug delivery systems. Wound dressings are primarily produced from natural and synthetic polymers. In this review, we focused on chitosan and cellulose nanocrystals as biomaterials for the development of hydrogels for wound management.

### 4.1. Gelatin

Gelatin is a natural biopolymer consisting of biologically active polypeptides derived from collagen in animal skin, bones, and other tissues. This polymer, being nontoxic due to its unique chemical and physical nature has been investigated as wound dressings and drug delivery systems [37]. Gelatin is also biocompatible, promotes cell adhesion and growth, non-immunogenic substrate of matrix metalloproteinases, and cost economy. Gelatin polymer consists of a large number of glycine, proline, and 4-hydroxyproline residues, which can have either acidic or basic properties depending on the extraction method [36]. Anionic acidic gelatin is useful for the delivering of positively charged bioactive agents whereas cationic basic gelatin is useful as drug system for negatively charged bioactive agents, forming polyion complexes. Its gelling properties can be controlled by chemical crosslinking with crosslinkers such as glutaraldehyde and genipin, that has been widely used for the development of wound dressings and as controlled release drug delivery systems. Gelatin has excellent property to form films, and thus is suitable material to produce capsules with rapid dissolution in gastric fluids. It is highly hydrophilic and has good swelling properties. Gelatin-based scaffolds have been used for a variety of biomedical applications, such as bone regeneration, skin tissue engineering [37], nerve tissue engineering, cardiac tissue engineering, tubular scaffolds, wound dressing and drug delivery systems [38]. Its application in drug delivery systems and wound healing is limited by poor mechanical properties. This disadvantage is overcome by the incorporation of other natural and synthetic polymers to reach the desirable biocompatibility and at the same time to have improved the mechanical and physical properties of nanofibers [38,39].

### 4.2. Cellulose

**Figure 2:**
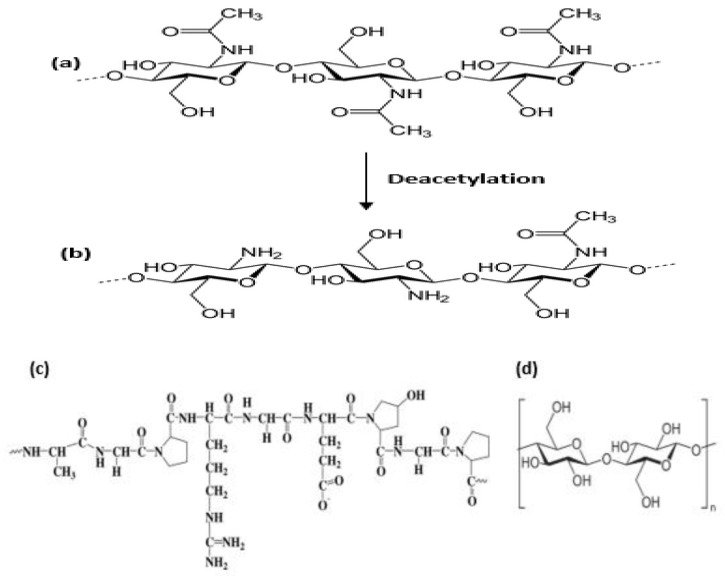
Polymer structure of (**a**) Chitin (**b**) Chitosan (**c**) Gelatin (**d**) Cellulose.

Cellulose is the most abundant natural polymer on earth, being the main structural component of plant cell walls. Cellulose has excellent characteristics, including recyclability, tunable surface features (Figure 2d), less risks of toxicity, biodegradability, biocompatibility [40]. Three types of nanocellulose are known, namely bacterial nanocellulose, cellulose nanocrystals, and cellulose nanofibers [41]. Bacterial nanocellulose is used for antibacterial wound healing and can safely and effectively improve wound healing [42]. Cellulose nanocrystals are excellent biomaterials with tunable surface chemistry. Recently, several studies have been focused on the topic of modification of cellulose nanocrystals, such as by esterification, oxidation [43], carbamation, amidation, etherification [44]. In the past years, it has been reported that cellulose nanocrystals can be oxidized with periodate and form several aldehyde groups [45]. The oxidation of cellulose with periodate leads to C2 and C3 carbon bond cleavage and aldehyde functional group formation on these carbon atoms. Therefore, dialdehyde cellulose nanocrystals may react with the free amino groups from chitosan or gelatin same as glutaraldehyde. This type of reaction is widely known as the Schiff base reaction [44].

Zhang research group developed a well-reinforced chitosan/bacterial cellulose hydrogel, which demonstrated improved mechanical properties and bactericidal activity [46]. The in vivo study showed that the wound dressing with chitosan/bacterial nanocellulose was totally filled with new epithelial cells within a period of two weeks, with no significant side reactions.

### 4.3. Chitosan

Chitosan is a linear natural amino polysaccharide obtained by alkaline N-de acetylation of chitin (Figure 2a,b) commonly derived from exoskeleton of crustaceans such as crabs, shrimps and lobsters [22,47]. Chitosan and its derivatives are widely known due to their functionalities, being biocompatible, biodegradable, non-toxic, bio-adhesive, antimicrobial, antioxidant; and due to its wound healing properties is considered as an excellent material for wound dressings [42]. It can be used to form membranes, sponges, scaffolds and hydrogels. Hydrogel dressing due to the ability to provide optimal moist healing environment, can protect, interact, contract the wound, and facilitate wound healing [4]. Additionally, chitosan derivatives can easily be produced by chemical modification of hydroxyl- and amino-groups present in the biopolymer (Table 1). Some of these derivatives consist of N-carboxymethyl-, N-succinyl-, N-acyl-, N-carboxybutyl-, N-carboxyethyl-, 5-methylpyrrolidinone-, N-N-dicarboxymethyl-, O-succinyl-, and O-carboxymethyl-chitosan derivatives, etc. Chitosan has poor mechanical properties, and it can easily undergo deformation through external applied stress, but this challenge can be overcome by incorporating it with suitable polymers such as cellulose nanocrystals, to improve its mechanical properties for production of wound coverings [48].

Chitosan is a biopolymer that is soluble in dilute aqueous acidic medium at a degree of deacetylation of 50% and higher (which depends on the origin of the polymer) as a result of its primary amino groups that have a pKa value of 6.3. The solubility takes place by the protonation of the amino group (–NH_2_) of the D-glucosamine repeating unit, whereby the polysaccharide is changed to a polyelectrolyte in acidic media. Solubility of chitosan is commonly carried out in acetic acid by dissolving it in 1% or 0.1 M acetic acid [49]. Table 1 summarizes some of the common modifications of chitosan along with their principal properties [50].

## 5. Preparation of Chitosan/Cellulose Nanocrystals Hydrogels

**Figure 3:**
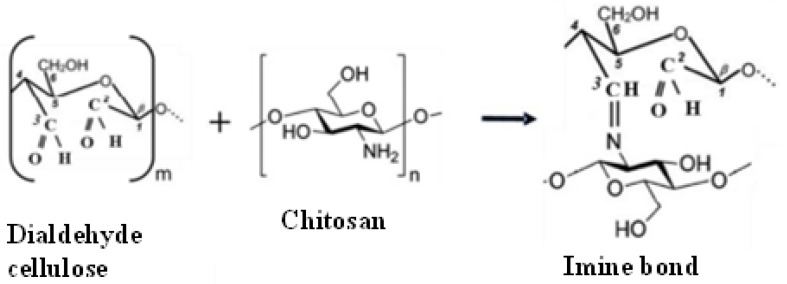
Formation of crosslinking between dialdehyde cellulose and chitosan.

Cellulose and chitosan have chemical similarities and biocompatibility of the polysaccharide structures, attracting great attention for their usage as composite biomaterials. Nanocelluloses due to their high mechanical properties, large surface area, and aspect ratio can be used as reinforcement in nanocomposites. Its incorporation in chitosan can also improve the mechanical properties and stability of chitosan-based composites [12]. The preparation of chitosan solution is commonly carried out by dissolution of chitosan in dilute acetic acid due to its poor solubility in water. Chitosan derivatives such as carboxymethyl chitosan is water-soluble when pH is greater than 7 [50]. The chitosan/cellulose hydrogel is formed by covalent linking of the chitosan polymer with cellulose nanocrystals where the bond formation is irreversible. The cross-linking of chitosan and cellulose polymers can be formed through the reaction of their functional groups (such as OH, COOH, and NH_2_) without any cross-linkers such as glutaraldehyde [49]. There are different approaches to chemically crosslinked chitosan with cellulose nanocrystals. The most used crosslinked technique of chitosan/cellulose nanocrystals is based on the oxidation of cellulose nanocrystals. The hydroxyl groups on the surface of cellulose can be selectively oxidized to carboxylic acid groups using TEMPO-mediated oxidation or to aldehydes using oxidizing agents such as periodate. In the case of carboxylic acid oxidation, the amino groups of chitosan will then react with carboxylic acid groups on oxidized cellulose nanocrystals using carbodiimide. For aldehyde modification, the amino groups of chitosan will then react with aldehyde groups through a Schiff base reaction (the formation of imine bonds) (Figure 3) [51], forming strong covalent bond without the use of any toxic chemical crosslinker, such as the commonly used glutaraldehyde.

## 6. Clinical Trials/Commercial Chitosan and Cellulose Wound Dressings

Currently, a number of cellulose and chitosan hydrogel wound dressings are under certain phases of clinical trials, and some of the dressings are marketed products as depicted in Table 2. The wound dressings are reported to be safe and effective for the management of different types of wounds [48,50].

## 7. Bioactive Hydrogel Wound Dressings

Modern hydrogel wound coverings should actively take part in the process of wound healing. Active wound dressings can be developed based on the encapsulation of bioactive components, including drugs, cells, growth factors and the wound dressing material. In any case, the desired wound dressing should actively assist in the wound healing process and should be cost-effective for clinical applications.

### 7.1. Bioactive Chitosan-Based Hydrogel

Wound dressing material can actively take part in wound healing. Hydrogels such as precursors derived from natural origin and derivatives affect wound healing. Biomaterials hydrogels such as gelatin, chitosan, alginate promote cell proliferation and migration, and growth and also enhance antibacterial activities [52,53,54,55]. Recent studies have proposed the usage of these biopolymers for different bioactive wound dressing applications. Murakami et al. employed a mixture of biopolymers consisting of chitin, fucoidan and chitosan to developed sheets of bioactive hydrogels that significantly boost tissue granulation and blood vesicles in non-healing wounds [56]. Moreover, chitosan polymer is mostly used to develop wound dressings because of its microbicidal, hemostatic properties. Recently, hydrogels such as chitosan/PEG, chitosan/PEG/poly(vinyl pyrrolidone) coated cotton fibers, chitosan/poly(vinyl alcohol), chitosan/poly(vinyl alcohol)/)poly-(ethylene oxide) hydrogels, carboxymethyl chitosan/gelatin, and chitosan-lactic acid have been demonstrated by researchers as suitable wound dressings promoting healing, in terms of the healing duration, degree of tissue granulation, production of collagen fibers, epithelialization and angiogenesis, and the inflammatory phase [57,58].

### 7.2. Drug Incorporated Hydrogel Dressings

Another promising properties of bioactive wound dressings are their ability for the prolong and/or controlled delivery of therapeutic agents. The loaded bioactive molecule can target several important places in wound healing. Pain killers such as aspirin, ibuprofen, lidocaine, acetaminophen, are mostly used in skin burns and wounds with high bacterial colonies or infection [53,59,60,61] (Table 3). Investigated hydrogel delivery systems for wound management are based on chitosan, polyvinyl alcohol and poloxamers and other biopolymers. For wounds with high bacterial infection, hydrogel dressings incorporated with antimicrobial agents are the most preferred and effective choice. Taking into account the increasing number of threats of antibiotic resistant bacterial strains, several studies, such as ciprofloxacin loaded in chitosan/alginate hydrogels, tetracycline loaded in alginate-cellulose nanocomposite hydrogel, and gentamicin incorporated in chitosan and sodium fusidate released from polyvinyl alcohol/poly vinylpyrrolidone/ propylene glycol hydrogels have been investigated [62,63,64].

Another procedure to prevent the growth of bacterial colonies is the application of microbicidal agents such as biological active peptides, metals naturally obtained compounds. Recently, comprehensive studies have been conducted on microbicidal activities of nanoparticles such as zinc oxide, silver and titanium dioxide nanoparticles. Zhao et al. developed a chitosan-based multifunctional hydrogel wound dressing containing in situ rapidly bioreduced silver nanoparticles with an excellent antibacterial properties which accelerated the healing process of infected wounds and promoted angiogenesis and collagen deposition [65]. Neibert group fabricated a microbicidal wound dressing by incorporating silver nanoparticles which was chemically crosslinked with alginate polymer [66]. Furthermore, silver nanoparticles promoted wound repair, increased the rate of healing, collagen deposition, formation of epithelia, improved the new tissues stability. Titanium dioxide nanoparticles incorporated in chitosan-pectin hydrogel and chitosan/polyvinylpyrrolidone dressing demonstrated a good microbicidal activity and at the same time improved the wound healing in animal models [67]. However, metals or metal oxide nanoparticles can be toxic to cells depending on the concentration. Recently, researchers have focused on the use of naturally derived antimicrobial agents, including essential oils, tea tree, and lemons incorporated in alginate wound dressing, melatonin loaded in chitosan-Pluronic^®^ F127 dressings and vanillin encapsulated in lysine-based dendrimers [68,69,70,71,72]. The main advantage of using hydrogels in the delivery of drugs is their ability to deliver drugs in a controlled release rate. This benefit can reduce drug dosages, costs and side effects, and therefore can enhance the therapeutic efficacy of their use [39].

**Table 3:** Summary of the application of drug-loaded chitosan hydrogels.

Drug	Preparation Technique	Potential Application	References
Gentamicin sulfate	EDC/NHS crosslinking	Anti-bacterial wound dressing	[63]
Apigenin	PEG-crosslinking	Diabetic wound dressing	[64]
Lupeol	Glutaraldehyde crosslinking	Wound dressing	[73]
Polyphenolic	Laccase crosslinking	Chronic wound dressing	[74]
Amoxicillin	Freeze–thaw	Antibiotic delivery	[75]
Ibuprofen	Not mention	Wound dressing	[76]
Tetracycline hydrochloride	Mixing	Scar preventive wound dressing	[77]
Tetracycline hydrochloride silver sulfadiazine	Casting/solvent evaporation	Anti-infection wound dressing	[78]
Superoxide dismutase	Polyelectrolyte complex	Antioxidant wound dressing	[79]

EDC: 1-ethyl-3-(3-dimethylaminopropyl)-carbodiiminde, NHS: N-hydroxysuccinimide. PEG: Polyethylene glycol.

### 7.3. Cells and Cell-Derived Peptides-Proteins Encapsulated in Hydrogels

Recently, bioactive hydrogels are receiving particular interest. Effective investigation of the complicated and dynamic wound healing process facilitated the recognition of different cell-derived peptides that mediate essential healing processes such as cell growth, migration and differentiation of endothelial cells [80,81]. The influence of the various growth factors such as fibroblast growth factor (FGF), epidermal growth factor (EGF), keratinocyte growth factor (KGF), platelet-derived growth factor (PDGF) and vascular endothelial growth factor (VEGF) have been studied [82,83,84,85,86]. Human EGF which was loaded in a heparin/polyethelene glycol scaffold or in an infrared responsive poly(N-isopropylacrylamide) hydrogel significantly promoted wound repair in mice, leading to high rate of cell granulation, re-epithelialization and growth of new blood capillaries [53,87,88]. However, the transdermal release and stability of growth factors had some limitations. In order to resolve this drawback, endothelial growth factor was entrapped in hyaluronic acid while conserving the biological properties of the growth factor [80,89]. Recent examples of bioactive dressings include fibroblast growth factor loaded in gelatin, keratinocyte growth factor encapsulated in chitosan-based hydrogel, chitosan/hyaluronic gels incorporated with nanoparticles of fibrin and vascular endothelial growth factor and some of created [90,91,92]. Currently, studies have shown that the encapsulation of two or more growth factors can produce a better result, because the healing process consists of different interactions between several growth factors [83]. The application of two or more growth factors surpasses the one growth factor administration by accelerating wound healing, higher rate of epithelialization, and the growth of new blood vessels. The most relevant ways are developing hydrogels with various growth factors for example the encapsulation of platelet-derived growth factor and vascular endothelial growth factor in chitosan/polyethylene oxide or wound dressings with platelet-rich plasma or platelet lysate [93]. Suitable delivery biomaterials for platelet rich plasma include chitosan, fibrin or gelatin [84,94,95]. Spanò research group described a biological active membrane of various blood plasma-derived components such as platelet-rich plasma combined with thrombin to treat skin ulcers [96]. A better approach to deliver several cell-derived peptides to a wound is the direct encapsulation of cells of interest in the hydrogel scaffold. This approach consists of stem cells, fibroblasts and keratinocytes from different biological sources [86]. Fibroblasts and keratinocytes are principally utilized to develop skin substitutes obtained from biodegradable scaffolds such as gelatin, alginate and chitosan [97,98,99]. Previous studies showed that the cellular delivery of keratinocytes, accelerating epithelialization, and vascular endothelial growth factor, promoting the development of new blood vessels, along with the release of cells to the wound area significantly enhanced the wound healing process [100]. The production and potential applications of wound dressings containing cells appear to be more promising in the near future. Stem cells can be self-renewed with the ability to differentiate into different types of cells depending on the milieu. Stem cells produce growth factors and cytokines which enables the cells to actively participate in the healing process [101]. The presence of stem cells has been reported to improve tissue granulation, accelerate angiogenesis and re-epithelialization, and collagen production and the rate of wound healing. However, the clinical application of stem cells in wound dressings are still under development due to high cost of growth factors, the reduction in therapeutic activity for long-time administration and inappropriate preservation, and high possibility of cancer as a result of extensive use of growth factors [102,103]. In addition, growth factors easily degrade at high concentrations of MMPs in chronic wounds, which requires the frequent changing of wound dressings (for example, twice a day for Regranex^®^ Gel) [86,104].

**Figure 4:**
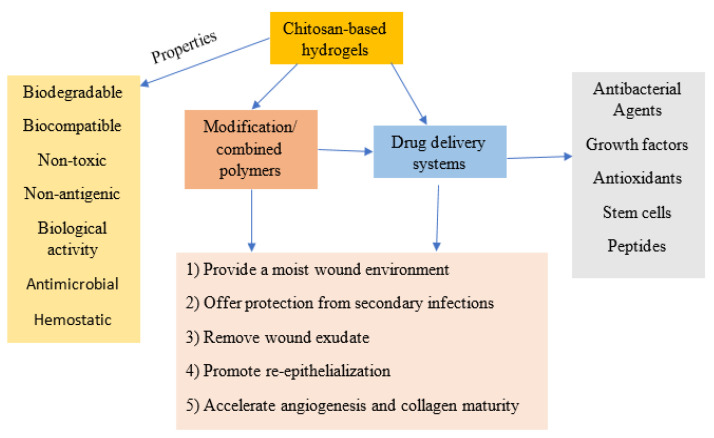
Summary of applications of chitosan-based hydrogels as both wound dressing and drug delivery systems.

In addition, chitosan matrix loaded with basic fibroblast growth factor (bFGF) in gelatin microparticles was investigated for the treatment of chronic ulcers of aged mice. The obtained results demonstrated that the hydrogel was an effective material for the delivery of growth factor and accelerated wound healing [105]. Chitosan/gelatin hydrogels demonstrated a positive effect on promotion of cell proliferation and angiogenesis, inducing granulation tissue formation, effectively prevents microorganisms, releasing bioactive agents, and accelerating the wound healing [50,106,107]. Figure 4 shows the summary of various applications of chitosan-based hydrogels as wound dressings and drug delivery systems.

## 8. Conclusions and Future Perspectives

Unique properties of hydrogels such as non-toxicity, biocompatibility, biodegradability, high water retention, soft texture, swelling properties, stimuli-responsive, controlled release of therapeutic agents and low cost are reason to consider them as most promising materials for wound management. Chitosan-based hydrogel is considered an excellent biomaterial due to its biodegradable, biocompatible, antimicrobial properties, and these properties could be modified by various natural or synthetic polymers. The capacity to release therapeutic molecules or growth factors to promote a more effective treatment is a necessary option for wounds. Chitosan-based hydrogels can deliver antibacterial agents, growth factors, stem cells, peptides, and other active substances in a prolong and controlled release fashion. This review covers the current state of wound dressing products with the main emphasis on chitosan and cellulose hydrogels as wound dressings.

The design, synthesis, and fabrication of hydrogels for wound dressing should be considered comprehensively, comprising multifunction, improvement of existing performances, stability of in all aspects, care impact for wound, and processability. We believe that the current challenges will be resolved, and hydrogel dressing will be a promising candidate for wound healing in near future with the continuous research in this field. Therefore, there is intensive research for the design and synthesis of advanced wound dressing materials with improved properties and they have to undergo clinical trials to ensure safety and effective wound treatment. It is expected that many hydrogels that are formulated from cellulose and chitosan will enter the clinical trials and market in the near future. Therefore, it can be concluded that chitosan-based hydrogels are highly explored and promising matrix for the use in drug delivery, wound dressing, and tissue engineering applications.

## Figures and Tables

**Table 1:** Some common modification of chitosan for wound healing dressings.

Modification	Properties
Carboxymethyl chitosan	Improved solubility in water. The commonly explored derivative of chitosan; it is amphoteric in nature and its solubility depends on pH, when the pH is >7 it is water soluble.
Thiolated urea derivatives	Thiourea chitosan boost the antibacterial properties.
Carbohydrate branched chitosan	Water soluble. Carbohydrate can be grafted on the chitosan backbone at the C2 position by reductive alkylation. They could be used for wound dressing and drug targeting.
Sugar derivatives	N-Succinyl chitosan is an amphoteric polymer consisting of amine, hydroxyl, and carbonyl groups. It has excellent physical, chemical, and biological properties as required in biomedical applications.
Alkylation chitosan	It is an essential amphiphilic polymer based on polysaccharides. Improves the stability of the interfacial films, promotes its solubility.

**Table 2:** Some chitosan and cellulose-based hydrogels in clinical trials/marketed product.

Hydrogels	Polymer	Characteristics	References
Chitoflex^®^ HemCon	Chitosan	Antibacterial and biocompatible. It adheres strongly to tissue surfaces and forms a flexible barrier.	[49]
Tegasorb^®^ 3M	Chitosan	Swells in the process of absorbing wound exudate and forming a soft gel. A sheet of waterproof Tegaderm^®^ film dressing covers the hydrocolloid. Good for leg ulcers and chronic wounds.	[3]
Chitopoly^®^ Fuji spinning	Chitosan	Good for developing antimicrobial wear, which helps to prevent dermatitis.	[50]
Chitoseal^®^ Abbott		It has good biocompatibility and hemostatic functions. Suitable for bleeding wounds	[50]
Chitopack C^®^ Eisai	Chitosan	Cotton-like chitosan. Fully repairs damaged body tissues and regenerate skin regularly.	[50]
FibDex^®^ (Nanofibrillar cellulose)	Cellulose	Efficiently heals wound at skin graft donor site, requires no dressing changes, self-detaches after re-epithelialization.	[48]
Bacterial nanocellulose	Cellulose	A great number of the patched skin did not show any symptom of edema and vesicles. It was non-irritant and safe.	[48]
Polyhexanide modified cellulose wound dressings	Cellulose	Clinical tests were performed on patients with pressure ulcers infected with Methicillin-resistant *Staphylococcus aureus.* The bacteria were completely eradicated.	[48]
Celox™		Rapid hemostatic property and reduces blood loss.	[48]
Chitoderm^®^ plus	Chitosan	Good absorbent properties.	[30,49]
Nanoderm™ Ag	Cellulose	Demonstrated a high degree of flexibility and prolonged antimicrobial properties. Effective for the treatment of infected wounds.	[48]

## Data Availability

The data presented in this study are available on request from the corresponding author.
